# The Surgery, Surgical Pathology and Surgical Anatomy of the Female Pelvic Organs

**Published:** 1880-09

**Authors:** 


The Surgery, Surgical Pathology, and Surgical Anatomy of the Female Pelvic Organs.—In a series of plates taken from nature, with commentaries, notes and cases. By Henry Savage, M.D., London, Fellow of the Royal College of Surgeons of England, etc. Third edition revised and greatly extended. 32 plates and 22 wood engravings, with special illustrations of the operations on Vesico-Vaginal Fistula, Ovariotomy and Perineal Operations. Wm. Wood & Co., New York. 1880.

We have just received through Mr. W. B. Dalston, this valuable addition to "Wood's Library of Standard Medical Authors." For the purpose for which it is intended it is unsurpassed and should be in the hand of every gynecologist.

